# Understanding splicing regulation through RNA splicing maps

**DOI:** 10.1016/j.tig.2010.12.001

**Published:** 2011-03

**Authors:** Joshua T. Witten, Jernej Ule

**Affiliations:** MRC Laboratory of Molecular Biology, Hills Road, Cambridge, UK, CB2 0QH

## Abstract

Alternative splicing is a highly regulated process that greatly increases the proteome diversity and plays an important role in cellular differentiation and disease. Interactions between RNA-binding proteins (RBPs) and pre-mRNA are the principle regulator of splicing decisions. Findings from recent genome-wide studies of protein–RNA interactions have been combined with assays of the global effects of RBPs on splicing to create RNA splicing maps. These maps integrate information from all pre-mRNAs regulated by single RBPs to identify the global positioning principles guiding splicing regulation. Recent studies using this approach have identified a set of positional principles that are shared between diverse RBPs. Here, we discuss how insights from RNA splicing maps of different RBPs inform the mechanistic models of splicing regulation.

## Studying alternative splicing using genome-wide approaches

Technological advances in the past decade have created the unprecedented ability to explore alternative splicing in a genome-wide manner. As the depth of analysis has increased, the estimated proportion of human genes that produce alternative mRNA isoforms has increased, from 35% in 1999 [1] to 94% in 2008 [2]. Splicing defects have been associated with many human diseases [3,4], and studies of the regulatory programmes that control splicing decisions have already revealed clues to the causes of several human diseases and identified splicing targets for RNA therapeutics [5,6]. Many diseases, however, might be affected by splicing regulatory errors in ways that have yet to be understood [7].

There are many ways to regulate alternative splicing. RNA–RNA interactions between distal sites are important for the regulation of mutually exclusive exons of the *Down syndrome adhesion molecule* (*Dscam*) transcript in *Drosophila melanogaster* [8]. This intricate regulation is particularly important because of the complex splicing of the *Dscam* transcript, which contains three clusters of 12, 48 and 33 mutually exclusive exons that can theoretically generate 38016 different alternative isoforms. A small molecule binding to an RNA riboswitch affects alternative splicing in the fungus *Neurospora crassa* by inducing changes in pre-mRNA structure [9]. Pre-mRNA interactions with noncoding RNAs, including a small nucleolar RNA [10] and an RNA related to 5S ribosomal RNA [11], have also been reported. Despite this potential diversity of regulatory mechanisms, protein–RNA interactions are considered the primary elements of splicing regulation and these interactions will be the focus of the remainder of this review.

Genome-wide studies play a key role in understanding the regulation of alternative splicing in disease and normal physiology. Initial bioinformatic studies have identified putative regulatory RNA motifs by comparing exons with different splice site strengths [12] or by comparing exons to pseudoexons [13]. Later studies have used the genome-wide data generated by splice-junction microarrays or RNA-seq to compare RNA motifs that are enriched near alternative exons with splicing patterns specific to tissues [2,14,15] or particular stages of differentiation [16,17] or disease [3,18]. Bioinformatic studies have also directly evaluated the importance of protein–RNA interactions in regulating splicing choices. This was achieved by analysing the presence of RNA motifs recognized by specific RNA-binding proteins (RBPs) near alternative exons. This approach was used to predict alternative exons regulated by serine/arginine-rich (SR), Nova and Fox proteins among others [19–22]. For instance, the evidence for the global role of Fox proteins in tissue-specific splicing regulation came from the enrichment of their binding motif (U)GCAUG near exons with brain or muscle-specific splicing patterns [2,14,23]. Similarly, the enrichment of this motif near exons with splicing changes in breast and ovarian tumours revealed a role for Fox proteins in human disease [3].

The pre-mRNA sequence elements required for *in vivo* splicing regulation have also been identified experimentally. Even though these elements most often map to intronic regions that are rapidly degraded upon splicing completion, they can be identified by the analysis of protein–RNA interactions using UV crosslinking and immunoprecipitation (CLIP; Box 1). CLIP data provided the first evidence for the global role of Nova proteins in brain-specific splicing regulation [24]. Below, we discuss the recent progress made by genome-wide studies and describe how combining protein–RNA interaction information with genome-wide splicing analyses can reveal global principles behind splicing regulation.

## RNA splicing map: an integrative approach to study splicing regulation

Early studies of splicing regulation indicated that SR proteins enhance exon inclusion, whereas heterogeneous nuclear ribonucleoproteins (hnRNPs) silence exon inclusion by antagonising the SR proteins [25]. This conclusion was supported by splicing minigene reporter studies of a small number of exons, which showed that SR proteins enhance exon definition by strengthening interactions between spliceosome components and the pre-mRNA, whereas hnRNPs block such interactions [26]. However, exceptions to this rule were found [26]. For instance, SR proteins normally enhance splicing by binding within the exon, but were found to repress splicing in the adenovirus L1 unit when bound to an upstream intron [27]. hnRNP L protein binding close to an alternative 5′ splice site led to silencing, whereas binding further downstream enhanced exon inclusion [28]. Similarly, Nova binding within an alternative exon led to silencing, but binding downstream enhanced exon inclusion [29]. These studies indicated that the positions of protein–RNA interactions play a major role in splicing regulation, although it was not known whether the position-dependent splicing effects were unique to each target RNA or if they represented general principles of splicing regulation that were common to different RNAs and, possibly, different RBPs.

To understand the general principles of splicing regulation, it is necessary to identify protein–RNA interactions with high resolution in a genome-wide manner. However, a single genome-wide data set rarely provides significant insight into splicing regulation on its own. Partly, this is because protein-binding sites are most often located far from alternative exons, as shown by the first analyses of Nova–RNA interactions using CLIP [24]. In addition, the RNA motifs recognised by RBPs are often degenerate and, therefore, expected to occur frequently in pre-mRNAs. For example, Nova proteins recognise the motif YCAY (Y represents either pyrimidine base) usually in clusters of multiple tetramers [30]. Many of these motifs can lead to high-affinity protein–RNA interactions that are nonfunctional. Furthermore, protein–RNA interactions are known to have roles in the regulation of other post-transcriptional processes, such as processing microRNA precursors or the 3′ end of mRNAs [31,32]. Therefore, analysis of genome-wide protein–RNA interactions alone is not sufficient to study the positional principles behind splicing regulation.

**Figure 1:**
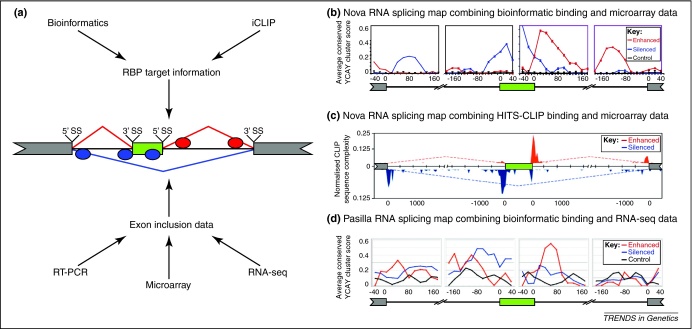
Schematic procedure for generating an RNA splicing map. **(a)** RNA splicing maps are generated by integrating RBP-binding data and splicing profiles. A variety of methodologies can provide this information depending on the model system, technology available and research goals. The simplest form of an RNA splicing map summarises the effects of RBP (ellipses) binding on a cassette exon. Exons are separated into those enhanced (red line) or silenced by the RBP (blue line), and control exons, which are not regulated by the RBP. To find positional principles of splicing regulation, RBP-binding data are combined for each group of exons into a hypothetical, composite cassette exon, with a focus on positions surrounding (typically within 200 base pairs) the 5′ and 3′ splice sites (5′ SS and 3′ SS), the potential branch points of the alternative exon (green box) and the flanking exons (grey boxes). **(b)** A Nova RNA splicing map for cassette exons generated by integrating the bioinformatic identification of Nova-binding sites and splice-junction microarray data (reproduced with permission from [20]). **(c)** A Nova RNA splicing map for cassette exons generated by integrating the HITS-CLIP experimental identification of Nova-binding sites and splice-junction microarray data (reproduced with permission from [32]). **(d)** An RNA splicing map for Pasilla, the *Drosophila* orthologue of Nova, generated by integrating the bioinformatic identification of Pasilla-binding sites and splicing profiles from RNA-seq data (reproduced with permission from [34]). As shown for the Nova RNA splicing maps in **(b)** and **(c)**, Pasilla-binding sites are most enriched within and immediately upstream of the skipped exons (blue line) and downstream of enhanced exons. The distance relative to the closest splice site is shown underneath each map. The position of RBP binding is shown on the *x*-axis. The frequency of RBP binding is shown on the *y*-axis in red for enhanced, blue for silenced and black for control exons.

The success of genome-wide studies lies in the integration of multiple, independent data sets [4]. Combining genome-wide protein–RNA interaction sites with the analysis of splicing profiles allows the analysis of RNA splicing maps, which determine the position-dependent regulatory effects of protein–RNA interactions (Figure 1a). Originally referred to as an ‘RNA map’ [20], the term ‘RNA splicing map’ is preferred here to distinguish it from analyses of the position-dependent regulation of other aspects of RNA processing. The initial approach used with Nova proteins combined bioinformatically identified Nova-binding sites with splicing profiles identified by splice-junction microarrays [20,33] (Figure 1b). A similar RNA splicing map was obtained later when protein–RNA interaction sites were determined experimentally by the high-throughput sequencing of CLIP [32] (Figure 1c). Moreover, instead of splice-junction microarrays, splicing profiles can now be derived from RNA-seq data [34] (Figure 1d). These three RNA splicing maps are not identical, which is expected because of the different methods used to derive them. However, in spite of this variability, the three maps detected the same primary positions of enriched Nova binding.

Studies of the polypyrimidine tract-binding protein (PTB) illustrate the need for independent protein–RNA interaction and splicing data sets when building RNA splicing maps. Initially, CLIP data were used to identify interaction sites and select target exons to collect splicing data by RT-PCR analysis. This RNA splicing map indicated that PTB primarily silences exon inclusion when bound downstream of exons [35]. When PTB-regulated exons were identified independently by microarray, the RNA splicing map indicated that PTB more often enhances exon inclusion when bound downstream of exons [36]. Thus, the integration of independent genome-wide data sets is required to identify general principles of splicing regulation.

## RNA splicing maps reveal general principles of splicing regulation

**Figure 2:**
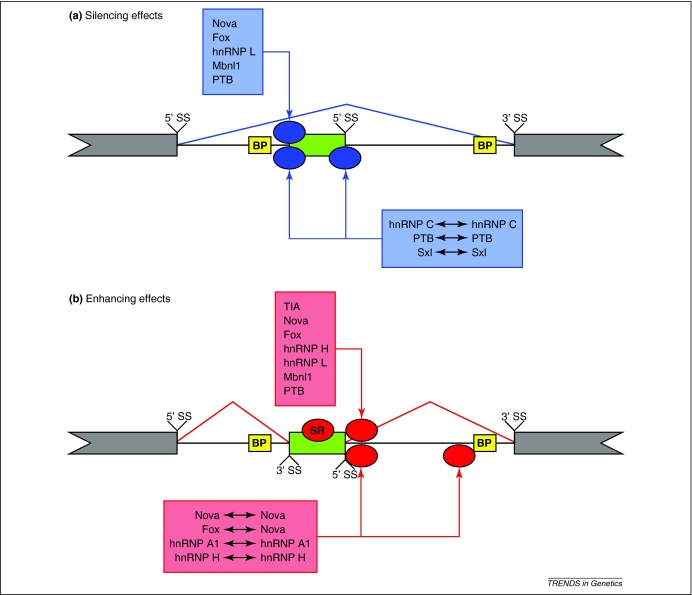
Shared positional effects identified by the RNA splicing maps of different RBPs. **(a)** Positions in silenced exons with the enriched binding of different RBPs. Shown above the transcript diagram, RBP (blue ellipse) binding to a single site close to 3′ SS silences exon inclusion (blue line). Even though not shown in this diagram, such a site can also lie within the exon or close to the 5′ SS. Shown below the transcript diagram, some RBPs bind at intronic positions close to 3′ and 5′ splice sites of the exon to efficiently silence exon inclusion. The arrows indicate that binding at the different positions is achieved by the different RBPs or a hnRNP C. **(b)** Positions of enhanced exons with the enriched binding of different RBPs. Shown above the transcript diagram, RBP (red ellipse) binding downstream of the 5′ splice site of a cassette exon promotes its inclusion (red line). Shown below the transcript diagram, RBP binding at multiple positions at both ends of the downstream intron enhances exon inclusion. The arrows indicate that interaction between the RBPs bound at both sides of the intron might be required for the enhancing effect. In contrast to the RBPs studied so far, SR proteins enhance inclusion when bound within exons. Upstream and downstream exons (grey boxes) and potential branch points (yellow boxes) are also indicated for positional reference.

The similarity of the RNA splicing maps of the mouse Nova and of Pasilla, the *Drosophila* orthologue of Nova, shows that position-dependent regulatory effects are highly conserved [34]. Common principles can be identified by comparing the RNA splicing maps of different RBPs that have been derived so far [18,20,21,32,35–41] (Figure 2). Surprisingly, a comparison of these RNA splicing maps reveals that RBPs share many common positional principles [42]. Nova, hnRNP C, L and H, Fox, PTB, and muscleblind (Mbnl1) silence exon inclusion by binding at positions close to the branch points, splice sites or within exons (Figure 2a). By contrast, Nova, hnRNP L, Fox, PTB, Mbnl1 and TIA proteins enhance exon inclusion by binding downstream of exons (Figure 2b).

The RNA splicing maps of hnRNP C and TIA proteins appear more restricted than other RBPs. hnRNP C exclusively silences exon inclusion when binding near the alternative exon (Figure 2a). By contrast, TIA proteins only bind downstream of exons (Figure 2b). What could be the reason for such restricted activity? hnRNP C binds RNA as a homo-tetramer and assembles into higher-order hnRNP particles on long RNAs [43]. Tetramer binding at multiple sites both upstream and downstream of the exon might allow the silencing of exon inclusion [40]. Silencing effects involving multiple binding sites was first observed for Sex-lethal and PTB using minigene reporters [44,45]. Binding at exon flanking sites has been shown to promote repressive RNA looping and interfere with interactions between the spliceosome components [46,47].

TIA proteins enhance exon inclusion when binding downstream of alternative exons, with no evidence for silencing when binding to other sites near the alternative exons [41]. Exclusive binding downstream of exons cannot be predicted from pre-mRNA sequences. Uridine-rich motifs, which TIA proteins bind with high affinity, are equally frequent upstream and downstream of exons [48]. TIA proteins interact with U1 small nuclear ribonucleoprotein (snRNP), the spliceosome component required for the recognition of the 5′ splice site and initiation of splice site pairing [49]. The yeast orthologue of TIA Nam8p is a core component of U1 snRNP [50]. The evolutionary conservation of TIA interaction with U1 snRNP suggests that the TIA–U1 snRNP complex might restrict TIA binding to positions downstream of exons. Taken together, the close association of hnRNP C and TIA proteins with defined higher-order complexes might be the reason for their more restricted position-dependent effects compared with other RBPs.

## The role of RBPs in intron definition

The *in vitro* and *in vivo* analysis of introns that require hnRNP A1 or hnRNP H for efficient splicing showed that these proteins need to bind both ends of the intron to stimulate splicing [51] (Figure 2b). Similarly, the Nova RNA splicing map indicated that Nova often enhances exon inclusion by binding at both ends of the downstream intron. In half of the exons with Nova binding immediately downstream of the exon, an additional Nova binding site is present close to the branch point of the intron, suggesting that the enhancing activity involves Nova binding the two sites at each end of the intron [20]. These effects can be explained by the intron definition model, which suggests that RBPs bound at both sides of an intron can interact to promote an RNA loop that brings the 5′ splice site in proximity to the branch point.

Heterotypic interactions between hnRNP A1 and hnRNP H could stimulate splicing by promoting RNA looping [52]. Similarly, conserved sequences around Nova-regulated exons suggest a functional interaction between the binding sites of Fox and Nova proteins when they bind to the two sides of an intron [22] (Figure 2b). These results indicate that homotypic and heterotypic interactions between RBPs bound to distal sites on pre-mRNAs might be a widespread mechanism for splicing regulation. As CLIP data for more RBPs become available, many more interactions between RBPs that regulate splicing by promoting pre-mRNA looping might be revealed.

## Global principles, specific mechanisms?

What do the common principles identified by RNA splicing maps tell us about the mechanisms of splicing regulation? The RBPs that share positional principles are not homologous to each other. Often they bind RNA with different domains such as KH in Nova, zinc finger in Mbnl and RRM in the other RBPs discussed above. The silencing effects, when binding near the branch point, splice sites or within the exon, could be explained by competition with core spliceosome components or SR proteins [20,25,26]. Explanation of the enhancing effects, when RBPs bind downstream of exons, is more challenging. These proteins might enhance exon inclusion by directly interacting with and stabilising U1 snRNP on the pre-mRNA. Alternatively, they might interact with TIA proteins to stabilize a TIA–U1 snRNP complex. They could also act by altering the RNA structure in a manner that increases the ability of the U1 snRNP to interact with the 5′ splice site. Lastly, as discussed above, they might form homotypic or heterotypic interactions that promote intron definition. Detailed biochemical studies will be required to unravel these mechanisms or possibly uncover new explanations for these shared positional principles.

Further analyses of RNA splicing maps and other RBPs are likely to identify global, position-based splicing effects that differ from those determined so far. For example, SR proteins enhance exon inclusion by binding within the exon [53], in contrast to the silencing effects of other RBPs (Figure 2b). Even though the binding sites for SR proteins are enriched in exons, they also bind to introns [19,54–56]. The RNA splicing maps of SR proteins could, therefore, provide new insight into their position-based splicing effects. Furthermore, given that homotypic and heterotypic protein–protein interactions can change the conformation of pre-mRNA to either silence or enhance exon inclusion [22,46,47,51,52], many combinatorial possibilities remain to be explored. Combinatorial RNA splicing maps might help identify new interactions between RBPs that regulate splicing through the modification of the pre-mRNA structure.

## Splicing effects of distal TIA binding suggest a role of splicing kinetics

Above, we discussed the splicing effects of RBP binding sites proximal to alternative exons. The analysis of CLIP data and RNA splicing maps also suggests that RBPs can regulate splicing when binding only at positions distal to alternative exons [20,24,32,35,40,41]. Indeed, the Nova RNA splicing map indicates that Nova proteins can silence the inclusion of an alternative exon when binding close to the preceding exon [20].

**Figure 3:**
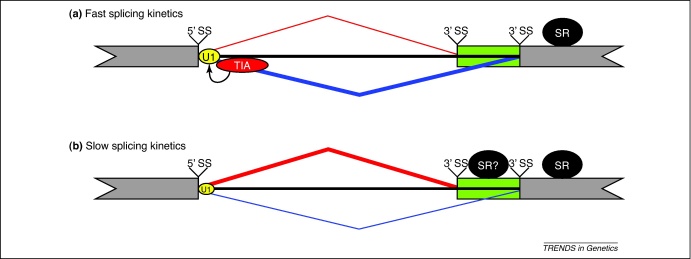
The distal regulation of variable-length exons by TIA proteins. **(a)** TIA protein (red ellipse) binding downstream of the 5′ splice site (5′ SS) stabilises U1 snRNP (yellow ellipse) binding, thereby potentially increasing the rate of transition from the H complex (RBP assembly) to A complex (splice site pairing), which we refer to as faster splicing kinetics. This reduces the time available for RBPs, such as SR proteins (black ellipse), to assemble on the nascent transcript, leading to the preferential exclusion (thick blue line) of the variable region (green box) relative to inclusion (thin red line). Local enhancement leads to distal silencing. **(b)** In the absence of TIA proteins, the reduced stability of U1 snRNP binding might slow splicing kinetics and thereby increase the temporal window for the assembly of RBPs on the nascent transcript. For example, the binding of additional SR proteins (marked by a question mark) could promote the use of an alternative 3′ splice site (3′ SS), resulting in the preferential inclusion (thick red line) of the variable region of the exon. The blue line represents exon skipping, red line represents exon inclusion and the thickness of the lines represents the extent of the splicing choice.

Recently, TIA proteins, like Nova proteins, were found to silence exon inclusion when binding to the preceding exon [41]. In addition to silencing the distal cassette exons, TIA proteins are also able to silence distal alternative 3′ splice sites by binding at the preceding exon (Figure 3), showing that TIA could achieve a distal splicing effect without modulating competition between multiple 5′ splice sites. One possible explanation for this distal effect is that TIA acts by affecting the kinetics of the splicing reaction. The splicing reaction proceeds through three stages before splice site pairing is committed [57]. In the first stage, referred to as H complex, RBPs assemble on the pre-mRNA. In the next stage, referred to as E complex, the U1 snRNP and SR proteins assemble on the splice sites and within the exons. In the third stage, referred to as A complex, the branch point is recognized by the second spliceosome component the U2 snRNP, which leads to splice site pairing and commitment to a specific splicing choice [57]. TIA proteins might increase the rate of transition from the H complex to the A complex by enhancing the U1 snRNP recruitment to the 5′ splice site of the preceding exon. This would restrict the temporal window available for other RBPs to assemble on the alternative exon and thereby influence the splicing choice. Because the effects of distal TIA binding are associated with silencing, such a model would suggest that this temporal window primarily affects the assembly of RBP complexes that enhance exon inclusion, such as SR proteins [41] (Figure 3).

## Integration of multiple variables into splicing decisions

Splicing decisions result from the integration of multiple variables, primarily changes in the expression of RBPs, changes in RBP activity because of signalling pathway-induced post-translational modifications [58] and changes in transcription or chromatin [59]. Promoter identity [60], transcriptional elongation [61] and chromatin modifications [62–64] can all affect the splicing of specific alternative exons. Splicing factors accumulate on nascent transcripts as the RNA polymerase synthesises the pre-RNA [65–67]. The comparison of splicing intermediates in the chromatin and nucleoplasm indicates that splicing is largely completed before the release of the transcript from the chromatin template [68]. These observations underlie several models to explain how the coupling of splicing to transcription and chromatin affects splicing choices.

The recruitment model proposes that certain splicing regulators, particularly those containing arginine-rich, positively charged regions (such as SR proteins), bind the hyperphosphorylated, negatively charged C-terminal domain of elongating RNA polymerase II (Pol II CTD) [69]. In support of this model, antibodies against Pol II CTD coimmunoprecipitate SR proteins. Furthermore, the localization of SR proteins to sites of transcription and the activity of certain SR proteins also require Pol II CTD [61,70]. Interestingly, studies have suggested that in addition to Pol II CTD chromatin might also play a role in recruiting RBPs. Specific modifications of histone 3 have been shown to recruit U2 snRNP components or PTB to nascent transcripts [64,71]. Whether Pol II CTD and chromatin recruit RBPs to the nascent transcripts in a sequence-independent manner, or only increase the local concentration of RBPs on chromatin, remains to be resolved.

Chromatin immunoprecipitation experiments have shown that the treatment of formaldehyde crosslinked cell lysate with RNase releases SR proteins from the *FOS* gene [70]. This indicates that interactions between SR proteins and Pol II are dependent on nascent RNA, rather than SR proteins being recruited to the RNA via interactions with Pol II. In such a case, an alternative model is necessary to explain how transcription modulates splicing. The first-come first-served model proposes that the speed of transcriptional elongation directly affects the splicing of cassette exons, such that slow elongation gives the upstream intron additional time to be spliced before the downstream intron becomes available [72]. This model was recently evaluated by analysing the splicing intermediates of fibronectin exon 33, where one flanking intron was spliced and the other remained unspliced [68,73]. This study showed that the downstream intron is spliced before the upstream intron, even in conditions of slow transcriptional elongation. Even though splicing intermediates do not necessarily reflect the order of intronic commitment to splicing [73], faster splicing of the downstream intron is not easily compatible with the first-come first-served model.

Finally, the splicing kinetics model proposes that the temporal window available for RBP assembly on nascent transcripts can affect splicing choice. This temporal window is determined by the kinetics of transition from the H to A splicing complexes. This model was first proposed to explain the effect of an elongation-defective mutant of RNA Pol II on the splicing of alternative 5′ splice sites in the adenovirus *E1a* gene [72]. Owing to the alternative 5′ splice sites being in close proximity to each other, their regulation could not be explained by the first-come first-served model. Instead, slower transcription elongation might allow more time for RBPs and splicing machinery to assemble on the nascent transcript before the 3′ splice site becomes available to pair with the 5′ splice sites [59,72]. As discussed earlier, this splicing kinetics model could also explain the ability of TIA proteins to modulate splicing choices from distal sites (Figure 3). Thus, the splicing kinetics model could explain the splicing effects of many different factors, as long as these factors can change the temporal window available for RBP complexes to assemble on the nascent transcript before the splicing choice is made.

## Asymmetric decision making in alternative splicing

**Figure 4:**
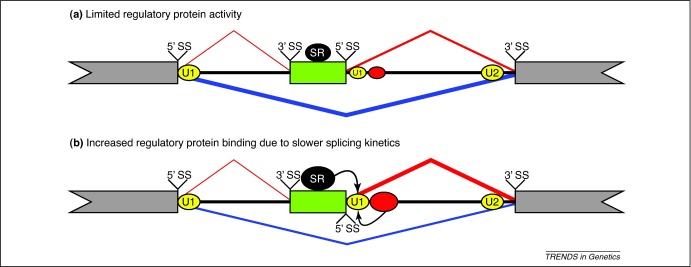
Asymmetric splicing decision making. Splicing decisions for cassette exons (green box) are the result of the exon skipping (blue line) pathway competing with two exon inclusion (red lines) pathways. RNA splicing maps indicate that RBPs often enhance splicing by binding to the downstream intron. As a result, these RBPs asymmetrically enhance the downstream intron removal pathway of exon inclusion. In this schematic example, RBP (red ellipse) assembly on the nascent transcript promotes the pathway where the downstream intron is spliced first. **(a)** In the absence of sufficient RBP activity, perhaps because of fast splicing kinetics reducing the temporal window for RBP assembly, the exon skipping pathway dominates the competition. **(b)** Slower splicing kinetics might allow the increased binding of SR proteins (black ellipse) to the exon, and other RBPs downstream of the exon that promote stable U1 snRNP (yellow ellipse) binding to the 5′ splice site (5′ SS). In this case, increased RBP assembly enhances exon inclusion by promoting the removal of the downstream intron (red line).

To conceptualise how RBP binding at different positions affects splicing decisions, we can consider the splicing decision as a competition between commitment to three possible splicing pathways: exon skipping, upstream intron splicing and downstream intron splicing. The upstream and downstream intron splicing pathways both lead to exon inclusion. Splicing intermediates in *Nova1*–/– *Nova2*–/– brains have shown that the splicing effects of Nova proteins are restricted to the intron containing the Nova-binding sites [20]. Importantly, the intron containing the Nova-binding site is generally spliced first, indicating that RBP binding can create asymmetry in the exon inclusion pathways. RNA splicing maps indicate that protein–RNA interactions in the vicinity of alternative exons most often enhance downstream intron splicing or silence upstream intron splicing (Figure 2). This asymmetric effect could create a restricted competitive situation for alternative cassette exons, where only the downstream intron splicing competes with the exon skipping pathway (Figure 4a). In situations of such restricted competition, RBP binding would be necessary to promote exon inclusion by enhancing the downstream intron splicing pathway (Figure 4b).

Asymmetric splicing decision making could play a role in the previously described models. For instance, slow transcriptional elongation promotes the splicing of the downstream intron of fibronectin exon 33 [68,73]. According to the splicing kinetics model, slow transcriptional elongation might extend the temporal window for the assembly of RBPs on the nascent transcript. Thus, the observed asymmetric splicing effect on the fibronectin exon 33 downstream intron splicing could be a result of the asymmetric binding preferences of RBPs (Figure 4b). The analysis of splicing intermediates in the chromatin fraction has indicated that introns flanking fibronectin exon 33 were spliced faster than introns flanking either constitutive exons or two other alternative exons [68]. It remains to be seen if the fast splicing kinetics of flanking introns is a general characteristic of exons that are sensitive to the effects of distal RBP binding or transcriptional elongation.

## Concluding remarks

We have discussed how understanding the global principles behind the regulation of alternative splicing can provide insights into the mechanisms of splicing regulation. To date, RNA splicing maps have only been determined for individual RBPs. It is clear, however, from individual alternative exons that their splicing is regulated by multiple RBPs acting either competitively or cooperatively [74,75]. The high resolution of iCLIP (Box 1) makes this method particularly suited for combinatorial studies of multiple RBPs [40]. Building combinatorial RNA splicing maps to identify relations between the interactions of multiple proteins with proximal sites on the same RNA and under conditions that influence these regulatory interactions could greatly increase our understanding of combinatorial splicing regulation across the genome.

The development of RNA splicing maps from the combination of genome-wide protein–RNA interaction data with splicing profiles has allowed broad, position-based principles for splicing regulation to be inferred for a few RBPs. As the resolution and quantitative capacity of these methods improves with the development of iCLIP and RNA-seq, the ability to define these rules becomes more precise. At the same time, precise definition of these rules will help identify exons and RBPs that deviate from the basic splicing models. These deviations will help us better understand how multiple variables contribute to the regulation of splicing. To understand such integrated regulation, RNA splicing maps will need to be combined with analyses of other variables that contribute to alternative splicing decisions, such as splicing kinetics, transcriptional elongation speed, chromatin, the post-translational modifications of RBPs, RNA structure and the interactions of pre-mRNA with other noncoding RNAs. Finally, biochemical and kinetic studies of the insights gained from genome-wide analyses will be required to fully understand the mechanisms of splicing regulation.

## Box 1 Methods using UV crosslinking for genome-wide studies of protein–RNA interactions

**CLIP** (UV crosslinking and immunoprecipitation): Exposure to UVC light creates a covalent bond between proteins and the RNA to which they are bound. This physical link is used to isolate the RNAs bound by a specific protein using immunoprecipitation and denaturing gel electrophoresis. The protein is then digested, and the RNA is prepared for sequencing using the sequential ligation of two RNA adapters to prepare the cDNA library [24]. The short length of CLIP cDNA sequences is perfectly compatible with high-throughput sequencing and is referred to as HITS-CLIP (high-throughput sequencing CLIP) or CLIP-seq [32,38,42,76]. Unlike standard CLIP, PAR-CLIP (photoactivatable ribonucleoside-enhanced CLIP) incorporates 4-thiouridine into RNAs to generate protein–RNA crosslinks using UVA light, which has been claimed to increase crosslinking efficiency [76]. Related methods have been developed for situations in which it is not possible to isolate the native protein because of the lack of a specific antibody or if individual protein domains need to be studied. These methods include the affinity purification of proteins in yeast (UV crosslinking and cloning – CRAC) [77] and the affinity purification of tagged proteins in cell lines (UV crosslinking and affinity purification – CLAP) [41].

**iCLIP** (individual nucleotide resolution CLIP) isolates RNAs that are crosslinked to specific proteins as in standard CLIP. After protein digestion, an amino acid (or a short peptide) remains at the RNA crosslink site, which leads to the frequent truncation of cDNAs previously exploited to map crosslink sites using primer extension assays [78]. Using cDNA circularization to prepare the cDNA library allows for the high-throughput sequencing of cDNAs that truncate at that peptide [40]. Sequencing truncated cDNAs identifies the protein–RNA crosslink site with high resolution. The use of random barcodes to mark cDNAs during library preparation makes it possible to determine if identical sequences arose from multiple independent cDNAs or are the result of PCR amplification. These barcodes, therefore, help preserve the quantitative nature of the cDNA library.

## Glossary

Alternative splicing: The production of mature mRNAs that varies in sequence composition because of the use of different splices during the maturation of each transcript. Resulting transcripts can differ by the inclusion of cassette exons or terminal exons, usage of alternative splice sites of variable-length exons or intron retention.
Branch point: A splicing signal located in the intron upstream of the 3′ splice site. It contains an adenine, which is ligated to the 5′ splice site ribonucleotide to form the intron lariat.
Cassette exon: An exon that is included or skipped as one unit during alternative splicing.
Isoform: A mature mRNA representing one of multiple variations that can be created by alternative splicing.
RNA-seq: A method to analyse the transcriptome by reverse transcribing mRNAs into a cDNA library, which is sequenced by high-throughput sequencing. If the number of sequencing reads is sufficient, it can be used to evaluate alternative splicing.
Pre-mRNA: An RNA transcript as it is synthesised by RNA polymerase II, before intron removal via RNA splicing.
RNA riboswitch: An RNA sequence that changes its structure upon the binding of specific, small molecules. The change in RNA structure most often plays a role in regulating the expression of the resident mRNA.
Splice-junction microarrays: A microarray specifically designed to assay alternative splicing by probes (short sequences) that anneal to alternative exons and splice junctions.
5′ splice site: Highly conserved sequence at the 5′ end of the intron. During the first step of splicing, it undergoes nucleophilic attack from the 2′-OH of the branch point to form the intron lariat.
3′ splice site: Highly conserved sequence at the 3′ end of the intron. During the second step of splicing, it undergoes nucleophilic attack from the 3′-OH of the 5′ exon, leading to the release of the intron and the joining of the two exons.
